# *β*-Lactoglobulin Heptapeptide Reduces Oxidative Stress in Intestinal Epithelial Cells and Angiotensin II-Induced Vasoconstriction on Mouse Mesenteric Arteries by Induction of Nuclear Factor Erythroid 2-Related Factor 2 (Nrf2) Translocation

**DOI:** 10.1155/2019/1616239

**Published:** 2019-11-12

**Authors:** Giacomo Pepe, Manuela Giovanna Basilicata, Albino Carrizzo, Simona Adesso, Carmine Ostacolo, Marina Sala, Eduardo Sommella, Marco Ruocco, Stella Cascioferro, Mariateresa Ambrosio, Simona Pisanti, Veronica Di Sarno, Alessia Bertamino, Stefania Marzocco, Carmine Vecchione, Pietro Campiglia

**Affiliations:** ^1^Department of Pharmacy, University of Salerno, Fisciano, Italy; ^2^PhD Program in Drug Discovery and Development, University of Salerno, Fisciano, Italy; ^3^IRCCS Neuromed, Loc. Camerelle, Pozzilli, Italy; ^4^Department of Pharmacy, University of Naples Federico II, NA, Italy; ^5^Dipartimento di Scienze e Tecnologie Biologiche, Chimiche e Farmaceutiche (STEBICEF), University of Palermo, PA, Italy; ^6^Department of Medicine and Surgery, University of Salerno, Baronissi, Italy; ^7^European Biomedical Research Institute of Salerno, SA, Italy

## Abstract

Peptides derived from buffalo dairy products possess multiple healthy properties that cannot be exerted as long as they are encrypted in parent proteins. To evaluate the biological activities of encrypted peptide sequences from buffalo ricotta cheese, we performed a simulated gastrointestinal (GI) digestion. Chemical and pharmacological characterization of the digest led to the identification of a novel peptide endowed with antioxidant and antihypertensive action. The GI digest was fractionated by Semiprep-HPLC, and fractions were tested against reactive oxygen species (ROS) release in an H_2_O_2_-treated intestinal epithelial cell line. UHPLC-PDA-MS/MS analysis revealed the presence of an abundant *β*-lactoglobulin peptide (BRP2) in the most active fraction. Pharmacological characterization of BRP2 highlighted its antioxidant activity, involving ROS reduction, nuclear factor erythroid 2-related factor 2 (Nrf2) activation, and cytoprotective enzyme expression. The bioavailability of BRP2 was evaluated in intestinal transport studies through a Caco-2 cell monolayer. Equal bidirectional transport and linear permeability indicate that BRP2 was absorbed mainly through passive diffusion. In addition to its local effects, the BRP2 administration on mouse mesenteric arteries was able to reduce the angiotensin II-induced vasoconstriction by the Nrf2 nuclear translocation, the reduction of the active form of Ras-related C3 botulinum toxin substrate 1 (Rac1), and the NADPH oxidase activity. These data further highlight the role of buffalo ricotta cheese-derived peptides against oxidative stress-related diseases and suggest their health-promoting potential.

## 1. Introduction

Food proteins are an important source of bioactive peptides. These are inactive, since encrypted in their parent sequences, but turn active when released by fermentation or ripening during food processing or by digestive enzymes during gastrointestinal transit [1, 2]. Once released, the bioactive peptides are able to exert various physiological effects beneficial for human health [3]. In particular, bioactive peptides can either have local effects on the digestive tract or be absorbed through the intestine, playing a physiological role in tissues [4]. These peptides can exhibit various biological activities, such as antioxidant, antimicrobial, immunomodulatory, antithrombotic, and antihypertensive, depending on their amino acid sequence [5]. The size of active sequences may vary from two to twenty amino acid residues, and several peptides are known to reveal multifunctional properties since some regions in the primary structure of parent protein, considered “strategic zones,” contain overlapping sequences [6].

The effect of natural antioxidant peptides on health by treatment and prevention of numerous diseases is of great interest nowadays due to their safety, small size, low toxicity, and high activity in addition to the negative consumer perception about synthetic drugs [7]. This is why food-derived antioxidant peptides have become an interesting target in food chemistry. Enhancement of the body's antioxidant defense mechanism through dietary supplementation would seem to be a practical approach to reduce the level of reactive oxygen species (ROS) [8–10].

ROS are produced in a well-regulated manner to help maintain homeostasis at the cellular level in the normal healthy tissues, play an important role as second messengers, and regulate cellular function by modulating signaling pathways [11]. An imbalance in the equilibration of prooxidant/antioxidant status determines oxidative stress, characterized by damage to cellular macromolecules such as DNA, proteins, and membrane lipids, by human aging, and by diseases, such as gastrointestinal (GI) and cardiovascular pathologies [12].

The GI tract is prone to ROS attack as it is accessed by the outside environment with dietary factors that, together with resident immune cells and intestinal flora, are potential sources of ROS.

ROS have been linked with various inflammatory GI disorders such gastroesophageal reflux disease, gastritis, enteritis, colitis, and associated cancers as well as pancreatitis and liver cirrhosis [13]. Several studies demonstrate that oxidative stress also plays an important role in the pathogenesis and development of cardiovascular diseases, including hypertension, dyslipidemia, diabetes mellitus, atherosclerosis, myocardial infraction, angina pectoris, and heart failure [14]. In fact, oxidative stress is considered to be the main cause of endothelial dysfunction leading to cardiovascular complications, mostly through the reduction of nitric oxide (NO) bioavailability, which is one of the most important mediators of the physiological properties of endothelial cells. The increased production of ROS and decreased NO bioavailability promote endothelial dysfunction, leading to remodeling, platelet aggregation, loss of vasodilation, inflammation, and smooth muscle cell growth [15]. An imbalance between NO and ROS has been observed in patients with hypertension [16].

In order to prevent and counteract some GI pathologies and cardiovascular diseases, the employment of natural antioxidant molecules is crucial. Dairy products and their fractions can be considered carriers for the delivery of antioxidant peptides. We recently evidenced the antioxidant properties of buffalo milk dairy products and in particular buffalo ricotta cheese [17, 18].

In addition, several studies showed the antihypertensive effect of whey protein as renin-angiotensin-converting enzyme inhibitors, direct stimulators of endothelial NO, opioid receptor agonists, or direct inhibitors of endothelin-1 production, but no studies were described concerning the hypotensive activity of buffalo whey protein-derived peptides [19–22].

In this regard, the aim of the present work was to investigate the release, the intestinal absorption, and the biological activities of potential antioxidant peptides after simulated oral intake of buffalo ricotta cheese. After *in vitro* gastrointestinal digestion, the sample was separated into two fractions that were challenged for its antioxidant properties. The peptidomic workflow led to the identification of an abundant *β*-lactoglobulin peptide in the most active fraction. The effect of this peptide on oxidative stress induced by H_2_O_2_ in the intestinal epithelial cells (IEC-6) and by angiotensin II in mouse mesenteric arteries was evaluated, together with its bioavailability.

## 2. Materials and Methods

### 2.1. Preparation and Fractionation of Buffalo Ricotta Gastrointestinal Digest by Semiprep-RP-HPLC

The simulated gastrointestinal digestion of buffalo ricotta cheese was performed according to Pepe et al. [23]. Briefly, the lyophilized sample was incubated with pepsin at 37°C for 2 h to pH = 2, and the reaction was stopped by heating the solution at 95°C for 15 min. Then, the gastric digest was incubated with pancreatin, chymotrypsin, and bile salts at 37°C for 2 h to pH 7.5, and the reaction was stopped bringing the solution to pH 2.

The peptides released after gastrointestinal digestion of buffalo ricotta cheese were fractionated by semipreparative reversed-phase liquid chromatography. For the separation, a Shimadzu Semiprep-HPLC was employed consisting of two LC-20 AP pumps, a SIL-20 AP autosampler, a fraction collector FRC-10 A, a UV detector SPD-20 A equipped with a preparative cell, and a system controller CBM-20 A.

The separation was carried out on a Kinetex™ C18 column (150 × 21.2 mm × 5 *μ*m (100 Å)), and flow rate (20 mL min^−1^), injection volume (5 mL (2 mg mL^−1^)), detection UV (214 and 220 nm), and the collection were based on UV-triggering signal. The optimal mobile phase consisted of (A) H_2_O and (B) ACN both acidified by trifluoroacetic acid 0.1% (*v*/*v*). Analysis was performed in gradient elution as follows: 0.01-5.00 min, isocratic to 1% B; 5-40.00 min, 1-35% B; 40-43.00 min, 35-95% B; and 43-46.00 min, isocratic to 95% B, and then five minutes for column reequilibration. The fractions were collected on the basis of their elution times and thus hydrophobicity. In detail, the fractionation of peptide digesta led to the collection of two different aliquots: fraction I (BRF1), from 10.00 min to 20.00 min, and fraction II (BRF2), from 20.00 to 30.00 min.

### 2.2. Peptide Identification in the BRF2

Analyses of the bioactive peptides contained in the BRF2 were performed on a Shimadzu Nexera UHPLC system coupled online to an LCMS-IT-TOF mass spectrometer through an ESI source (Shimadzu, Kyoto, Japan). Separation of BRF2 was carried out on an Aeris™ Peptide XB-C18 column (100 × 2.1 mm × 1.7 *μ*m) (Phenomenex, Bologna, Italy). The flow rate and the column oven temperature were set to 0.5 mL min^−1^ and 60°C, respectively. The chromatograms were monitored at 214 and 220 nm. The mobile phase for the analysis of BRF2 consisted of 0.1% (*v*/*v*) HCOOH/H_2_O (A) and 0.1% (*v*/*v*) HCOOH/ACN (B). Analysis was performed in gradient elution as follows: 0.01-45.0 min, 0-30% B; 45-47.00 min, 30-95% B; and 47-49.00 min, isocratic to 95% B, and then five minutes for column reequilibration.

MS detection was operated in ESI^+^ mode, and MS/MS experiments were conducted in data-dependent acquisition; precursor ions were acquired in the range 300-2000 *m*/*z*.

A free trial of PEAKS 7.5 software (Bioinformatics Solutions Inc., Waterloo, Canada) was employed for sequence determination. A search was performed using a database search tool, by searching against the SwissProt/UniProt database (database *Bubalus bubalis* release 2017).

### 2.3. Synthesis and Quantification of Buffalo Ricotta Peptide 2 (BRP2)

Synthesis of the analogue peptide was performed according to the solid phase approach using standard Fmoc methodology, with a Biotage Initiator+Alstra (Uppsala, Sweden) automated microwave synthesizer (for detailed conditions, see Supporting Information [Supplementary-material supplementary-material-1]).

The quantification of BRP2 in buffalo ricotta digesta and BRF2 was performed on a Nexera UHPLC system coupled online to an LCMS-8050 mass spectrometer (Shimadzu, Kyoto, Japan), equipped with an ESI source operated in positive mode. MS/MS analysis was conducted in selected reaction monitoring (SRM), employing the synthetic peptide as an external standard. Stock solution was prepared in water, the calibration curve was obtained in a concentration range of 0.1-125 *μ*g L^−1^ with eight concentration levels, and triplicate injection of each level was run. Peak areas of BRP2 were plotted against the corresponding concentrations. Linear regression was used to generate the calibration curve (*y* = 0.0004*x*–1.5321) with *R*^2^ values being ≥0.9998 (see Supporting Information [Supplementary-material supplementary-material-1]).

### 2.4. IEC-6 Cells: Culture, Treatment, and Viability Assay

The IEC-6 cell line (CRL-1592), derived from normal rat intestinal crypt cells, was purchased from the American Type Culture Collection (ATCC, Rockville, MD, USA).

These cells were cultured by using Dulbecco's modified Eagle's medium (DMEM) (4 g/L glucose), supplemented with 10% (*v*/*v*) heat-inactivated foetal bovine serum, 1.5 g/L NaHCO_3_, 2 mM L-glutamine, and 0.1 unit mL^−1^ bovine insulin. Cells were used, for the experiments, between the 17^th^ and 21^st^ passages.

The IEC-6 cells (2 × 10^4^) were plated into 96-multiwell plates and allowed to adhere. After 24 h, cells were exposed to BRF1 and BRF2 (50-1.25 *μ*g mL^−1^) and BRP2 (100-1 *μ*M), for 24 h. Cell viability was then assessed using the MTT assay, as previously reported [24].

#### 2.4.1. Measurement of Intracellular ROS Release

ROS levels were evaluated by means of the probe 2′,7′-dichlorofluorescin-diacetate (H_2_DCF-DA) [25]. For this experiment, IEC-6 cells were plated into 24-well plates (8 × 10^4^ cells/well). After adhesion time of 24 h, cells were then treated with BRF1 and BRF2 (50-1.25 *μ*g mL^−1^) and with BRP2 (100-1 *μ*M), for 1 h, either alone or in the presence of H_2_O_2_ (1 mM) for further 1 h.

IEC-6 cells were then collected, and a PBS buffer was used in order to wash them. Subsequently, cells were incubated in PBS containing H_2_DCF-DA (10 *μ*M), for 15 min at 37°C. A fluorescence-activated cell sorter (FACSscan; Becton Dickinson, Franklin Lakes, NJ, USA) was used for the purpose of measuring cell fluorescence, and CellQuest software (Becton Dickinson, Milan, Italy) was employed in order to analyze it.

#### 2.4.2. Immunofluorescence Analysis for Nuclear Factor-Like 2 Activation

IEC-6 cells (2 × 10^5^ cells/well) were seeded on coverslips in a 12-well plate and treated with BRP2 at concentration of 50 *μ*M for 1 h, both alone and in the presence of H_2_O_2_ (1 mM) for further 1 h in order to evaluate nuclear factor- (erythroid-derived 2) like 2 (Nrf2) activation. After the cellular treatment, 4% paraformaldehyde in PBS was used to fix the cells. Then, IEC-6 cells were permeabilized with 0.1% saponin in PBS. After the blocking made with BSA and PBS, cells were incubated with a rabbit anti-Nrf2 antibody (Santa Cruz Biotechnology, Dallas, TX, USA) for 1 h at 37°C. The slides were then washed three times with PBS. After that, a fluorescein-conjugated secondary antibody (FITC) was added for further 1 h. 4′,6-diamidine-2′-phenylindole dihydrochloride (DAPI) was used for the counterstaining of nuclei. At the end, coverslips were mounted in mounting medium. Fluorescent images were taken under the laser confocal microscope (Leica TCS SP5, Leica, Wetzlar, Germany) as previously reported [26].

#### 2.4.3. Measurement of Heme Oxygenase 1 (HO-1), NAD(P)H Quinone Dehydrogenase 1 (NQO1), and Superoxide Dismutase (SOD) Expression

IEC-6 cells were plated into 96-well plates (1 × 10^4^ cells/well) and allowed to adhere. After 24 h, cells were treated with BRP2 (100-1 *μ*M) for 1 h, either alone or in the presence of H_2_O_2_ (1 mM) for further 1 h. After cellular treatment, IEC-6 cells were collected, washed with PBS, and incubated in fixing solution for 20 min and then in Fix Perm Solution for further 30 min. Anti-heme oxygenase 1 (Santa Cruz Biotechnology, Dallas, TX, USA), anti-NAD(P)H quinone dehydrogenase 1 (Santa Cruz Biotechnology, Dallas, TX, USA), or anti-superoxide dismutase (Santa Cruz Biotechnology, Dallas, TX, USA) antibodies were then added. The cells were then treated with the secondary antibody. A fluorescence-activated cell sorter (FACSscan; Becton Dickinson, Franklin Lakes, NJ, USA) was used for the purpose of measuring cell fluorescence, and CellQuest software (Becton Dickinson, Milan, Italy) was employed in order to analyze it.

### 2.5. *In Vitro* Intestinal Transepithelial Transport Studies

#### 2.5.1. Caco-2 Cell Monolayer Permeation Experiments

The colorectal adenocarcinoma (Caco-2) cell line was purchased from ATCC (Rockville, MD, USA). Cells were maintained in high-glucose DMEM (4.5 g/L) supplemented with 2 mM L-glutamine and 10% (*v*/*v*) heat-inactivated foetal bovine serum. Cells were cultured at 37°C in a humidified 5% CO_2_ atmosphere. To induce enterocytic Caco-2 differentiation, cells were seeded in a 12-well multiwell in transwell inserts (PET membrane, 0.4 *μ*m pore size, 1.12 cm^2^ surface area) at 2.6 × 10^5^ cells/cm^2^ and maintained for 21 days in complete medium. The medium was changed every second day. By 21 days, the monolayers become completely differentiated.

The integrity of the monolayers was evaluated by measurement of the transepithelial electrical resistance (TEER) using an EVOM2 epithelial voltohmmeter (World Precision Instruments, Sarasota, FL, USA). Only monolayers showing TEER higher than 300 *Ω* × cm^2^ were then used for transport experiments. The integrity of the monolayers was checked before, during, and after the experiment. The filters were washed for 15-20 min at 37°C adding prewarmed Hank's balanced salt solution buffered with 25 mM HEPES and NaHCO_3_ (0.35 g/L) at pH 7.4 to the apical (0.4 mL) and to the basolateral (1.2 mL) transwell compartments, as previously described [27]. For transport experiments, donor solution containing BRP2 peptide at the desired concentration (100-1 *μ*M) was added to the apical compartment for the apical to basolateral (absorptive) direction. Samples from the receiving compartment were collected at different time points up to 120 min (15, 30, 60, 90, and 120 min). Samples from the donor compartment were collected at time 0 and at the end of the experiment (120 min) for the calculation of the mass balance.

Samples were stored at −20°C until UHPLC-MS/MS analyses to measure the concentration of BRP2 in both compartments (for detailed conditions, see Supporting Information [Supplementary-material supplementary-material-1]).

The apparent permeability coefficient (*P*_app_) was calculated as described according to

(1) $$\begin{array}{c} P_{\text{app}}=\frac{{dM}_{{\text{R}}^{\left(t\right)}}}{dt}\times \frac{1}{A\times C_{\text{D}0}}, \end{array}$$

where *M*_R_ is the amount of substance in the receiver chamber, *A* (cm^2^) is the surface area of the barrier, and *C*_D0_ (*μ*M) is the initial donor concentration. The reduction in donor concentration was also taken after every sampling (see Supporting Information [Supplementary-material supplementary-material-1]) [28].

#### 2.5.2. Immunofluorescence Analysis on Caco-2 Cell Monolayers

The transwell membranes from TEER experiments were washed with PBS and fixed with 4% paraformaldehyde (PFA) for 15 min. Membranes were then washed in PBS and blocked with blocking solution (0.1% Triton, 1% BSA, 0.02% sodium azide, and 50 mM ammonium chloride) for 20 min at room temperature in the dark. Afterwards, they were incubated with an anti-zonulin 1 antibody (#402200, Invitrogen, Thermo Fisher Scientific, Waltham, MA, USA) at the final concentration of 2 *μ*g mL^−1^ at room temperature for 2 hours. Immunofluorescence staining was obtained by incubating the membranes for 90 min with Alexa Fluor 488 donkey anti-rabbit IgG (#A31573) at the final concentration of 4 *μ*g mL^−1^ (Invitrogen). The nuclei were counterstained with DAPI (1 : 2000). Membranes were cut down with an operating knife blade along the margin of the chamber and were mounted on slides using VectaMount solution (AQ Vector Laboratories, Burlingame, CA, USA). Slides were examined under a Nikon fluorescence inverted microscope (Nikon Instruments Europe, Firenze, Italy) and then analyzed through ImageJ software as previously described [29].

### 2.6. Vascular Reactivity Studies

Second-order branches of the mesenteric arterial tree (internal diameter between 150 and 250 *μ*m) were dissected and mounted on a wire myograph as previously described [30]. Briefly, vessels were equilibrated for 60 min at 45 mmHg intraluminal pressure in warmed oxygenated (95 : 5%, air : CO_2_) Krebs solution (pH 7.4) containing the following (mmol L^−1^): 120 NaCl, 25 NaHCO_3_, 4.7 KCl, 1.18 KH_2_PO_4_, 1.18 MgSO_4_, 2.5 CaCl_2_, 0.026 EDTA, and 5.5 glucose. Media and lumen diameters were measured with a computer-based video imaging system (Danish Myo Technology). Endothelium-dependent and endothelium-independent relaxation was assessed by measuring the dilatory responses to cumulative doses of acetylcholine (Ach, 10^−9^ to 10^−5^ mol L^−1^) or nitroglycerine (Nitro, 10^−9^ to 10^−5^ mol L^−1^), respectively, in vessels precontracted with phenylephrine (10^−9^ to 10^−5^ mol L^−1^). After evaluation of basal vascular function, we have tested the effect of peptide on angiotensin II-induced vasoconstriction (Ang II, 10^−9^ to 10^−5^ mol L^−1^), preincubating the vessels with different dosages of BRP2 (100-1 *μ*mol L^−1^).

#### 2.6.1. Dihydroethidium (DHE) Staining

DHE was used to evaluate the levels of oxidative stress in mouse mesenteric arteries as previously described [31]. Briefly, vessels were stained with 5 mol L^−1^ DHE for 20 min, then mounted and observed under a fluorescence microscope (Zeiss, Oberkochen, Germany). Images were acquired by a digital camera system.

#### 2.6.2. NADPH Oxidase Activity Measurement

NADPH oxidase (NOX) activity in a pool of mesenteric arteries was measured in untreated cells and cells treated with angiotensin II and preincubated with BRP2 plus Ang II as previously described [31]. In another experimental set, we measure NADPH oxidase activity in IEC-6 cells following the same protocol but using 150 *μ*g of protein extract. Vessels were placed in a chilled modified Krebs/HEPES buffer. Periadventitial tissue was carefully removed, and the vessels were repeatedly washed to remove adherent blood cells. A 10% vessel homogenate was prepared in 50 mmol L^−1^ phosphate buffer containing 0.01 mmol L^−1^ EDTA. The homogenate was then subjected to low-speed centrifugation (1000 g) for 10 min to remove unbroken cells and debris. 20 *μ*L was added to glass scintillation vials containing 5 *μ*mol L^−1^ lucigenin in 1 mL phosphate buffer. The chemiluminescence that occurred over the ensuing 5 min in response to the addition of 100 *μ*mol L^−1^ NADPH was recorded (Beckman LS6500 Multipurpose Scintillation Counter; Beckman Coulter, Fullerton, CA). In preliminary experiments, homogenates alone without the addition of NADPH gave only minimal signals. Furthermore, NADPH did not evoke lucigenin chemiluminescence in the absence of homogenate.

#### 2.6.3. Immunoblotting and Nuclear/Cytoplasmic Fractionation

Immunoblots were performed as previously described [32]. Briefly, 30 *μ*g tissue extract for each sample was separated by SDS-PAGE and transferred onto a nitrocellulose membrane. Blocked membranes were incubated with primary antibodies in TBS-Tween and 5% milk overnight. Blocked membranes were then incubated with anti-MnSOD (1 : 1500) and anti-*β*-actin (1 : 1000).

Nuclear and cytoplasmic fractions, obtained as previously described [32], were separated by SDS-PAGE and transferred onto nitrocellulose membranes [32]. Blocked membranes were incubated with anti-Nrf2 (1 : 2000), anti-GAPDH (glyceraldehyde 3-phosphate dehydrogenase, 1 : 3000), and anti-HDAC2 (histone deacetylase 2, 1 : 2000) overnight and then detected using an appropriate horseradish peroxidase-coupled secondary antibody (Millipore, Milan, Italy) and visualized with enhanced chemiluminescence. The purity of nuclear and cytoplasmic fractions was confirmed using anti-HDAC2 and anti-GAPDH, respectively. Immunoblotting data were analyzed using ImageJ software (developed by Wayne Rasband, National Institutes of Health, USA) to determine OD of the bands. The OD reading was normalized to account for variations in loading.

#### 2.6.4. Ras-Related C3 Botulinum Toxin Substrate 1- (Rac1-) GTP Pull-Down Experiments

Mesenteric arteries were lysed in a buffer containing NP-40 equipped with kit STA-401-1 (Cell Biolabs Inc., San Diego, CA). The p21-binding domain of p21-activated protein kinase bound to agarose beads was added, and active Rac1, binding to PAK1, was separated by repetitive centrifugation and washing. Then, the specimens were boiled in Laemmli buffer and subjected to SDS-PAGE, and Rac1 was quantified by immunoblot analysis. In detail, Rac1-GTP was detected with the monoclonal antibody anti-Rac1-GTP c (1 : 800; STA-401-1, Cell Biolabs Inc.) and total Rac1 with monoclonal anti-Rac1 (1 : 1000; Abcam). The amount of Rac1-GTP was normalized to the total amount of Rac1 in tissue lysates for the comparison of Rac1 activity (GTP-bound Rac1) among different samples.

### 2.7. Data Analysis

Data were reported as mean ± standard error mean values, of at least three independent experiments, each in triplicate. In order to analyze the effects of our treatments on increasing doses of acetylcholine, we performed a 2-way repeated measures ANOVA with the Bonferroni post hoc test for multiple comparisons. Statistical analysis was performed by analysis of the variance test, and multiple comparisons were made by the Bonferroni test. A *p* value less than 0.05 was considered significant.

## 3. Results

### 3.1. Antioxidant Effect of BRP2 on ROS Release in IEC-6 Cells Treated with H_2_O_2_

**Figure 1:**
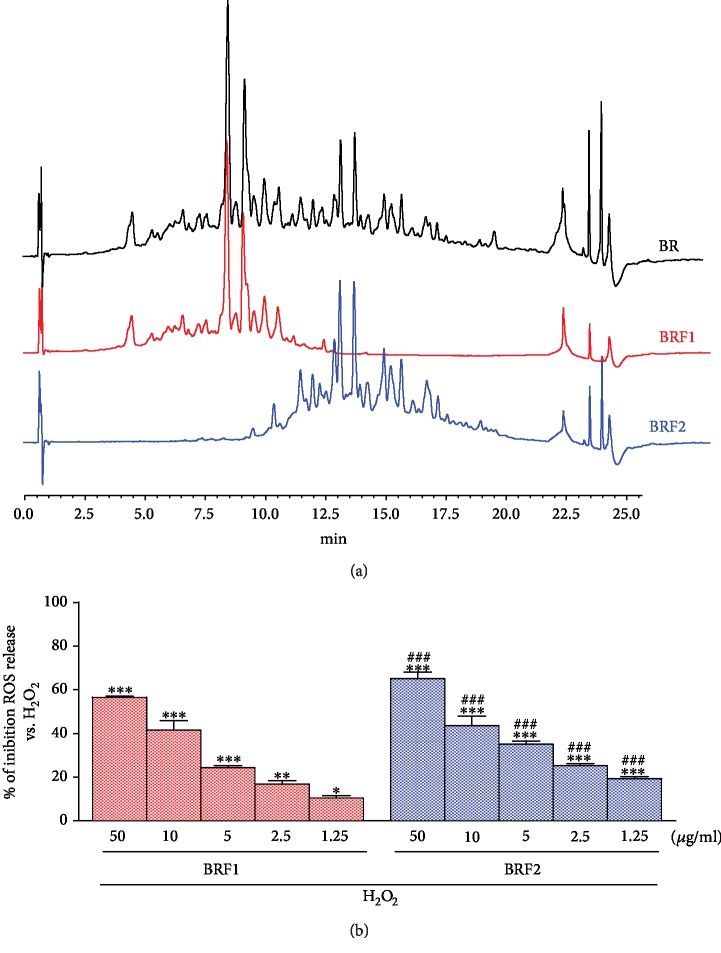
(a) Chromatographic profiles (*λ*: 214 nm) of the gastrointestinal digest of BR (black line), BRF1 (red line), and BRF2 (blue line). (b) Effect of BRF1 and BRF2 fractions on ROS formation, in IEC-6 cells, evaluated by H_2_DCF-DA. Values, mean ± s.e.m., are expressed as % of inhibition of ROS release vs. H_2_O_2_. ∗∗∗, ∗∗, and ∗ denote *p* < 0.001, *p* < 0.01, and *p* < 0.05 vs. H_2_O_2_, respectively. ### and ## denote *p* < 0.001 and *p* < 0.01 vs. BRF1+H_2_O_2_, respectively.

With the aim of investigating the potential of buffalo ricotta cheese against oxidative stress induced by H_2_O_2_ in IEC-6 cells, the intracellular ROS production was measured. The GI digest of buffalo ricotta cheese was separated into two different fractions BRF1 and BRF2 by Semiprep-RPLC (Figure 1(a)).

No cytotoxic effect was observed when IEC-6 cells were treated with BRF1 and BRF2 fractions (data not shown). On the other hand, both tested fractions significantly reduced ROS release in a concentration-dependent manner (*p* < 0.05 vs. H_2_O_2_; Figure 1(b)), with BRF2 fraction showing higher efficacy (*p* < 0.01 vs. BRF1; Figure 1(b)).

**Figure 2:**
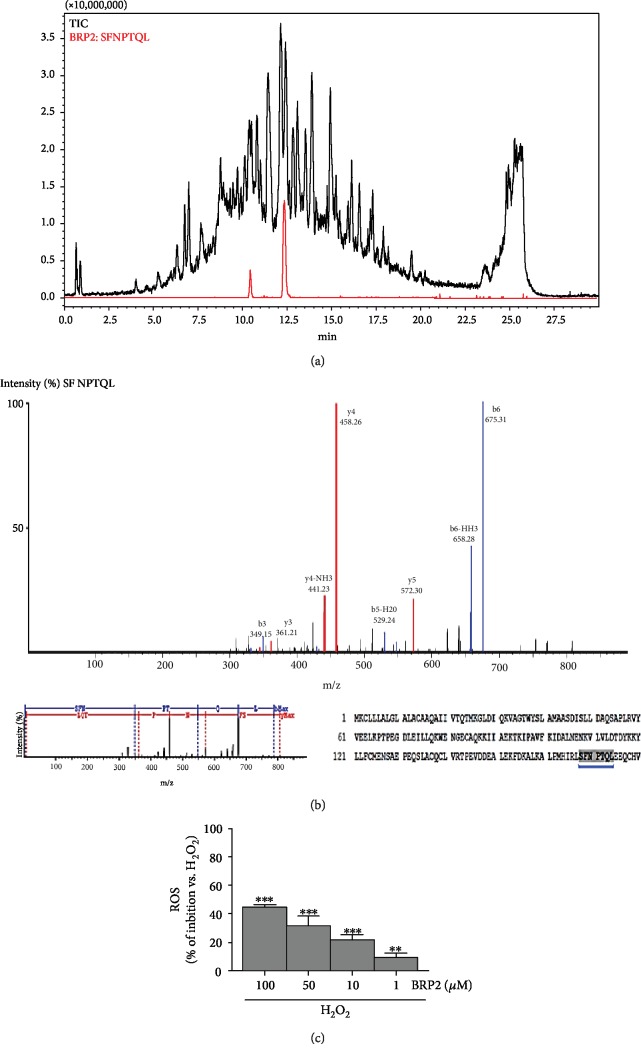
(a) Total ion chromatogram of BRF2 and (b) MS/MS fragmentation pattern of identified BRP2 (SFNPTQL) in BRF2 fraction. (c) Effect of BRP2 on ROS formation in H_2_O_2_-treated IEC-6 cells. Values, mean ± s.e.m., are expressed as % of inhibition of ROS vs. H_2_O_2_. ∗∗∗ and ∗∗ denote *p* < 0.001 and *p* < 0.01 vs. H_2_O_2_.

Thus, we focused on the identification of most abundant peptides of this fraction by UHPLC-PDA-MS/MS analysis. An intense peak in BRF2 was selected and identified as BRP2 (Figure 2(a)), namely, Ser-Phe-Asn-Pro-Thr-Gln-Leu (*β*-LG, f168-174, and SFNPTQL, Figure 2(b)). The relative amount of the peptide was calculated by MS/MS in 1 mg of BR digest and BRF2 (14.73 ± 0.38%*μ*M and 33.48 ± 0.56 *μ*M, respectively).

To investigate its biological properties, the peptide was synthesized by an Fmoc solid-phase approach (see Supporting Information [Supplementary-material supplementary-material-1]). Finally, the antioxidant potential of BRP2 was tested in IEC-6 cells treated with H_2_O_2_. Our results showed that BRP2 caused, at all tested concentrations (100-1 *μ*M), a significant decrement of ROS release induced by H_2_O_2_ (1 mM; *p* < 0.01 vs. H_2_O_2_, Figure 2(c)), thus exerting a cytoprotective effect against induced oxidative stress.

### 3.2. Evaluation of BRP2 Bioavailability

To assess BRP2 bioavailability, its transmembrane permeability was evaluated through Caco-2 fully differentiated cell monolayers [28]. As shown in [Supplementary-material supplementary-material-1] (see Supporting Information), the transport amounts of BRP2 increased approximately linearly, in a time- (0-120 min) and concentration-dependent (1-100 *μ*M) manner.

BRP2 showed moderate transport due to *P*_app_ values, ranging from 0.20 to 0.53 × 10^–6^ cm/s (see Supporting Information [Supplementary-material supplementary-material-1]). Finally, the *P*_app_ of BRP2 in the apical to basolateral direction (A-B), as well as that in the basolateral to apical direction (B-A), was compared to explore the possible transport mechanism. In particular, the efflux ratio, defined as the quotient of the secretory permeability and the absorptive permeability (B-A/A-B), was less than 1 suggesting that passive diffusion could be the main intestinal transport mechanism of BRP2 [33]. Moreover, in order to evaluate the effects of BRP2 on Caco-2 monolayer integrity and cell vitality, we performed immunofluorescence analysis on transwell inserts at the end of transport studies. As shown in [Supplementary-material supplementary-material-1] (see Supporting Information), Caco-2 cell monolayer integrity was preserved upon BRP2 treatment at all the concentrations tested as confirmed by tight junction protein zonulin-1 expression (green) and cell vitality.

### 3.3. BRP2 Reduces Angiotensin II-Induced Vasoconstriction and Oxidative Stress in the Mouse Mesenteric Artery

**Figure 3:**
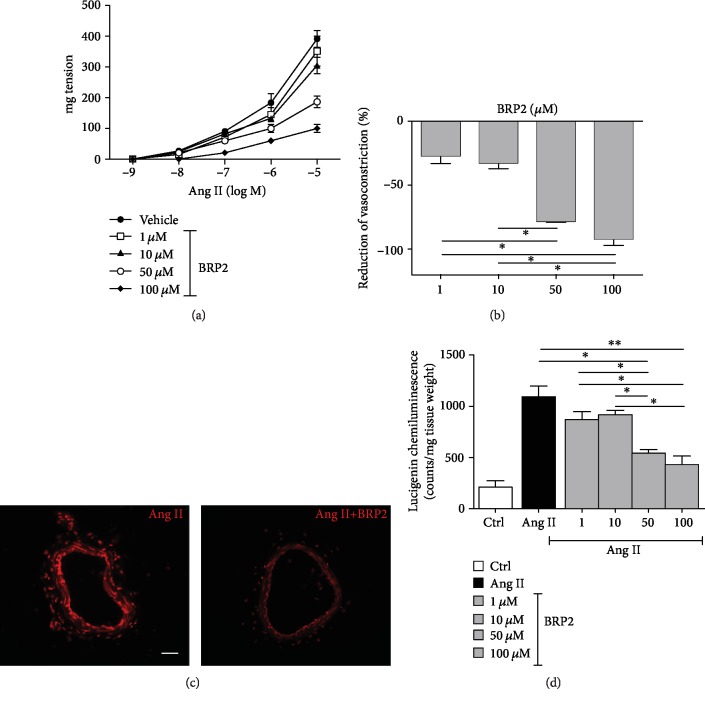
(a) Vascular responses to increasing doses of angiotensin II (10^−9^ to 10^−5^) of mouse mesenteric arteries preincubated with increasing doses of BRP2 (1, 10, 50, and 100 *μ*M). (b) Bar graph of the last time point of dose-response curve to angiotensin II (10^−5^ M). (c) In situ detection of superoxide generation with DHE staining in segments of mesenteric arteries treated with Ang II (10^−5^ M) alone or plus BRP2 (100 *μ*M). Scale bar: 50 *μ*m. (d) Graphs of superoxide production in mesenteric arteries measured continuously in the presence or absence of BRP2 by using 5 *μ*mol L^−1^ lucigenin-enhanced chemiluminescence. Values are mean ± s.e.m., expressed as RLU/(s·mg dry weight) (*n* = 4).

In order to investigate the antioxidant capability of BRP2 also in *ex vivo* model able to reproduce the cardiovascular condition of the vascular system, we performed experiments on the mouse mesenteric artery that is considered the prototype of resistance vessels involved in the modulation of systemic hemodynamic parameter. Interestingly, the preincubation of mesenteric arteries with increasing doses of BRP2 showed a progressive dose-dependent reduction of Ang II-induced vasoconstriction (Figure 3(a)), with maximal effects at 100 *μ*M, with a reduction of the Ang II-vasoconstrictive response of about 92.0 ± 4.0% (Figure 3(b)). This functional effect drove us to explore its action on the oxidative stress status, since oxygen-derived free radicals are selectively involved in the vascular response to Ang II. By DHE staining, we showed that BRP2 specifically reduces the Ang II-induced ROS production (Figure 3(c)). To support this effect, the measurement of NOX activity revealed that BRP2 is capable of markedly attenuating the lucigenin signal in a dose-dependent manner (Figure 3(d)).

### 3.4. Antioxidant and Hypotensive Effects of BRP2

The endogenous antioxidant system mainly consists of intracellular enzymatic antioxidants that are responsible for redox homeostasis balance. Nrf2 is an intracellular transcription factor that regulates the expression of several genes to activate antioxidative enzymes and detoxifying factors [34]. For these reasons, in order to give an insight into the molecular mechanisms underlying the antioxidant effects of BRP2, its influence on this specific antioxidant pathway was studied.

**Figure 4:**
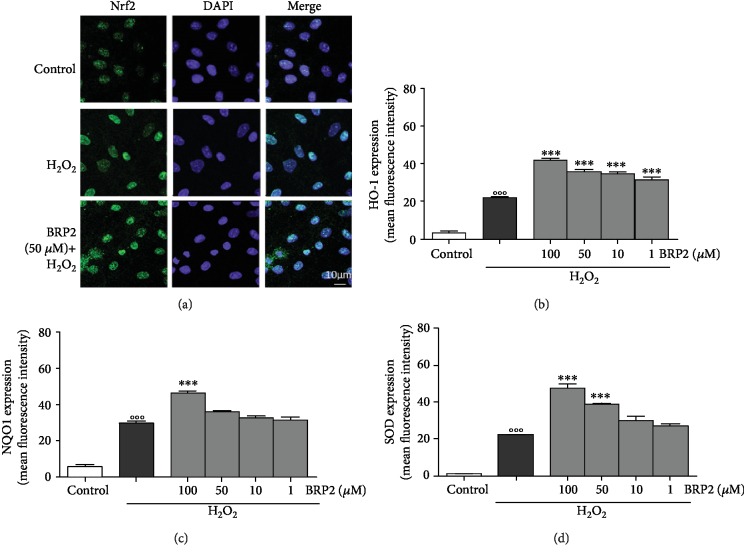
(a) Effect of BRP2 on Nrf2 nuclear translocation (scale bar: 10 *μ*m). Blue fluorescence and green fluorescence indicate localization of the nucleus (DAPI) and Nrf2, respectively. Effect of BRP2 on (b) HO-1, (c) NQO1, and (d) SOD expression in the IEC-6 cells, evaluated by the cytofluorimetric technique. Values, mean ± s.e.m., are expressed as % of inhibition of HO-1, NQO1, and SOD expression vs. H_2_O_2_. °°° denotes *p* < 0.001 vs. control. ∗∗∗ denotes *p* < 0.001 vs. H_2_O_2_.

**Figure 5:**
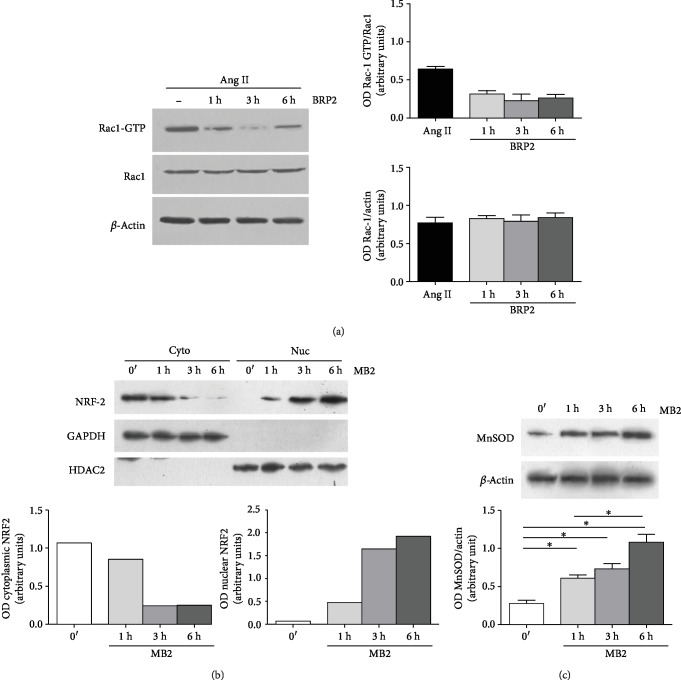
(a) Representative immunoblot from the pull-down assay of mouse mesenteric arteries for active Rac1 (Rac1-GTP). (b) Immunoblot analysis for Nrf2. Cytoplasmic (Cyto) and nuclear (Nuc) fractions were prepared from untreated mouse mesenteric arteries or treated with BRP2. GAPDH and HDAC2 were used as cytoplasmic and nuclear markers, respectively. Right: nuclear/cytoplasmic ratios for Nrf2 are plotted from densitometry (*n* = 3). (c) Representative immunoblot for MnSOD in mouse mesenteric arteries treated with BRP2 (100 *μ*M) and Ang II (10^−5^ M) (*n* = 3).

As shown in Figure 4(a), nuclear Nrf2 levels are increased in IEC-6 cells treated with BRP2 (50 *μ*M)+H_2_O_2_ (1 mM), with respect to H_2_O_2_ alone. It is known that Nrf2 activation leads to the expression of cytoprotective enzymes. In our experimental model, the effect of BRP2 on HO-1, NQO1, and SOD enzymatic expression was assessed. We observed that the expression of cytoprotective enzymes was significantly enhanced in the presence of H_2_O_2_ (1 mM; *p* < 0.001 vs. control). Administration of BRP2 (100-1 *μ*M) further increased HO-1 (*p* < 0.001 vs. H_2_O_2_; Figure 4(b)), NQO1 (*p* < 0.001 vs. H_2_O_2_; Figure 4(c)), and SOD expression (*p* < 0.001 vs. H_2_O_2_; Figure 4(d)). Nrf2 is generally held in the cytoplasm as an inactive complex bound to a repressor molecule and sensor of intracellular redox state. We found that in a time-dependent manner, BRP2 is able to induce Nrf2 translocation to the nucleus, where it turns active. Already starting from 1 hour of treatment, it is possible to appreciate the translocation of this factor that becomes maximal after 6 hours from BRP2 treatment (Figure 5(a)). Moreover, associated with the Nrf2 translocation, it was possible to note that at 1 hour there was an increase in MnSOD expression (Figure 5(b)).

It is well known that Ang II-induced ROS production is mainly mediated by NADPH oxidase activation, a multimeric complex that requires the small GTPase Rac1 to become active. Some studies have reported the functional and mechanistic connection between Rac1 and the transcription factor Nrf2. Based on these evidences, using the pull-down assay, we found a 50% reduction of Rac1-GTP after 1 hour of BRP2 treatment that further reduces up to six hours, thus supporting the capability of BRP2 to inhibit the angiotensin II-induced ROS production through NADPH oxidase recruitment inhibiting Rac1 activation (Figure 5(c)).

## 4. Discussion

In our previous study, the peptidomic profile of six different commercial dairy products based on buffalo milk was highlighted, revealing the presence of numerous peptides with immunomodulatory, antihypertensive, antioxidant, antimicrobial, anticancer, and antidiabetic properties [17]. However, only one-third of the identified peptides showed a recognized biological activity. Based on this data, we started a rational biological characterization of the six selected commercial products [18]. Buffalo ricotta cheese showed the highest antioxidant activity, compared to the other investigated buffalo dairy products. The peptidomic approach led to the identification of an abundant peptide, corresponding to the fragment 60-72 of *β*-lactoglobulin, namely, BRP, with interesting antioxidant activity [18]. With respect to the previous study, based on ultrafiltration with different cut-off membranes, in the present study, we fractionated the entire buffalo ricotta cheese digest by semipreparative liquid chromatography. Two main fractions were obtained in the most active fraction, and abundant *β*-lactoglobulin peptides (f168-174, SFNPTQL, and BRP2) were detected. The antioxidant potential of this peptide was not reported so far (see Supporting Information [Supplementary-material supplementary-material-1]); thus, we focused on its possible potential against oxidative stress, in particular on its ability to decrease ROS release. The intestine is the main organ of exposure and/or absorption of nutrients, toxic food contaminants, and metabolic products coming from the intestinal bacteria. The alteration of the integrity and function of the intestinal epithelium produces a negative impact on the rest of the body [35]. In many cases, the intestine responds adequately against the oxidative stress, but aging or disequilibrium in the redox state of the gut can induce intestinal pathologies such as inflammatory bowel disease, gastroduodenal ulcers, and colon cancer [36].

Our results showed that *β*-lactoglobulin-derived peptide BRP2 reduced ROS release induced by H_2_O_2_ in IEC-6 cells. Interestingly, BRP2 possessed a discrete bioavailability, showing a moderate absorption through a fully differentiated Caco-2 intestinal monolayer, without affecting its integrity and tight junction zonulin-1 protein expression.

To understand the antioxidant effect of BRP2 peptide, its molecular basis was investigated.

Nrf2 is a transcription factor that plays a central role in the regulation of antioxidant and phase 2 detoxifying enzymes and related proteins [37]. An increase in intracellular ROS enhances nuclear translocation of Nrf2 and expression of its target genes such as HO-1, NQO1, and SOD [38, 39]. Our results indicated that BRP2 protects intestinal epithelial cells from oxidative stress by ROS release inhibition and by upregulation of cytoprotective enzymes via the Nrf2/ARE pathway.

An oxidant mechanism of BRP2 could be related to the presence of amino acidic residues such as proline and threonine in its primary sequence as previously reported for *β*-lactoglobulin and *β*-casein peptides [18, 40].

A growing body of evidence indicates that an imbalance between endogenous reactive oxygen species and antioxidants in favor of the former contributes markedly to vascular dysfunction [41]. Based on the local antioxidant properties and on the moderate intestinal permeation of BRP2, we decided to investigate its potential systemic effects in an *ex vivo* mouse model of vascular reactivity. The most important endogenous bioactive octapeptide that exerts a potent vasoconstrictor through ROS production, modulating systemic hemodynamic parameters, is represented by Ang II. Our studies clearly demonstrated that pretreatment with BRP2 inhibits the Ang II-derived vasoconstrictive responses of mouse mesenteric arteries, in a dose-dependent manner. The 90% of inhibition after the exposure to the maximal dose of the peptide was obtained. This potent effect evoked by BRP2 is strictly related to its antioxidant properties, counteracting the oxidative stress induced by Ang II. In this regard, several evidences suggest that NAD(P)H oxidase is a major source recruited by Ang II to induce ROS generation in the vascular wall [42]. The evaluation of NADPH oxidase activity revealed a significant reduction of enzymatic activity after pretreatment with BRP2.

NOX is a multisubunit enzyme complex that requires specific interactions with a plethora of molecules. In this regard, the small GTPase Rac1 is essential for the correct assembly of NADPH subunits and their activation [43]. The treatment of mesenteric arteries with BRP2 significantly reduces Rac1 activation, supporting the effect of the peptide on the reduction of NADPH oxidase activity and the reduction of vasoconstrictive responses to Ang II. These *ex vivo* results demonstrate that BRP2 is able to act on two concomitant mechanisms, the reduction of the active form of Rac1 with a consequent reduction of NOX activity and the induction of nuclear translocation of Nrf2 that is pivotal in cellular defense against oxidative stress [44].

## 5. Conclusions

In conclusion, the results obtained highlight the important role of BRP2 in intestinal and cardiovascular protection, both inhibiting ROS release and enhancing an important antioxidant response consisting of Nrf2 pathway activation and cytoprotective enzyme expression. The antioxidant effects evoked in mice mesenteric arteries suggest BRP2 as a novel peptide candidate with promising cardiovascular effects and pave the way to its *in vivo* characterization in a model of cardiovascular disease.

## Data Availability

The data used to support the findings of this study are included within the article and the supplementary information files.

## Abbreviations

Ang II: Angiotensin II
BR: Buffalo ricotta
BRF: Buffalo ricotta fraction
BRP: Buffalo ricotta peptide
Caco-2: Colorectal adenocarcinoma
DAPI: 4′,6-diamidine-2′-phenylindole dihydrochloride
DHE: Dihydroethidium
GI: Gastrointestinal
GAPDH: Glyceraldehyde 3-phosphate dehydrogenase
H_2_DCF-DA: 2′,7′-dichlorofluorescin-diacetate
HDAC2: Histone deacetylase 2
HO-1: Heme oxygenase-1
IEC-6: Intestinal epithelial cell line
NO: Nitric oxide
NOX: NADPH oxidase
NQO1: NAD(P)H quinone oxidoreductase 1
Nrf2: Nuclear factor erythroid 2-related factor 2
Rac1: Ras-related C3 botulinum toxin substrate 1
ROS: Reactive oxygen species
*P*_*app*_: Apparent permeability coefficient
SOD: Superoxide dismutase.

## Supplementary Materials

Supplementary Materials Figure S1: (A) chromatographic profile acquired by RP-UHPLC-UV and (B) mass spectrum of synthetic BRP2 obtained by direct infusion Fourier-transform ion cyclotron resonance MS. Figure S2: transport of BRP2 across Caco-2 cell monolayer. Table S1: the apparent permeability coefficient (*P*_app_) values of BRP2 with different concentrations. Figure S3: fluorescence micrograph of the Caco-2 cell monolayers. Caco-2 cell monolayers treated with BRP2 (50 *μ*M, 2 h) from TEER experiments were stained for tight junction protein expression of zonulin-1 (FITC, green). Nuclei were counterstained with DAPI (blue). Pictures are representative of two independent experiments. Original magnification, 200x. Figure S4: (A) graphs of NADPH superoxide production in IEC-6 cells measured continuously in the presence or absence of BRP2 by using 5 *μ*mol L^−1^ lucigenin-enhanced chemiluminescence. Values are mean ± s.e.m., expressed as counts/mg proteins (*n* = 4). (B) Representative immunoblot of three independent experiments from the pull-down assay of IEC-6 cells for active Rac1 (Rac1-GTP), Ang II (10-5 M), and BRP2 (100 *μ*M) (*n* = 4).
