# Supplementary material for: β-Lactoglobulin Heptapeptide Reduces Oxidative Stress in Intestinal Epithelial Cells and Angiotensin II-Induced Vasoconstriction on Mouse Mesenteric Arteries by Induction of Nuclear Factor Erythroid 2-Related Factor 2 (Nrf2) Translocation
Source: Oxid Med Cell Longev. 2019 Nov 12;2019:1616239. doi: 10.1155/2019/1616239 (PMC6877959; doi:10.1155/2019/1616239)
Supplement: Supplementary Materials — Figure S1: (A) chromatographic profile acquired by RP-UHPLC-UV and (B) mass spectrum of synthetic BRP2 obtained by direct infusion Fourier-transform ion cyclotron resonance MS. Figure S2: transport of BRP2 across Caco-2 cell monolayer. Table S1: the apparent permeability coefficient (Papp) values of BRP2 with different concentrations. Figure S3: fluorescence micrograph of the Caco-2 cell monolayers. Caco-2 cell monolayers treated with BRP2 (50 μM, 2 h) from TEER experiments were stained for tight junction protein expression of zonulin-1 (FITC, green). Nuclei were counterstained with DAPI (blue). Pictures are representative of two independent experiments. Original magnification, 200x. Figure S4: (A) graphs of NADPH superoxide production in IEC-6 cells measured continuously in the presence or absence of BRP2 by using 5 μmol L−1 lucigenin-enhanced chemiluminescence. Values are mean ± s.e.m., expressed as counts/mg proteins (n = 4). (B) Representative immunoblot of three independent experiments from the pull-down assay of IEC-6 cells for active Rac1 (Rac1-GTP), Ang II (10-5 M), and BRP2 (100 μM) (n = 4). [file 1616239.f1.docx]

Supporting Information for the manuscript:

# β-lactoglobulin heptapeptide reduces oxidative stress in intestinal epithelial cells and Angiotensin II-induced vasoconstriction on mice mesenteric arteries by induction of Nuclear factor erythroid 2-related factor 2 (Nrf2) translocation

**Appendix S1. Solid phase synthesis of BRP2**

N^α^-Fmoc-protected amino acids, Fmoc-Leu-Wang resin, HBTU, HOAt, DIEA, piperidine and trifluoroacetic acid were purchased from Iris Biotech (Germany).

Peptide was synthesized on a Fmoc-Xaa-Wang resin (0.150 g, 0.69 mmol/g) previously deprotected with 25% piperidine/DMF (1×3 min, 1×10 min) at room temperature. The resin was then washed with DMF (4 × 4.5 mL). The following protected amino acids were then added on to the resin stepwise. Each coupling reaction was accomplished using a 3-fold excess of amino acid with HBTU and HOAt in the presence of DIEA (6 eq.) and were performed at 75°C for 10 minutes (2×). After each coupling step, the Fmoc protecting group was removed as described above. The resin was washed with DMF (4 × 4.5 mL) after each coupling and deprotection step. The N-terminal Fmoc group was removed, the resin was washed with DCM (7×) and the peptide was released from the resin with TFA/TIS/H_2_O (90:5:5) for 3 h. The resin was removed by filtration, and the crude peptide was recovered by precipitation with cold anhydrous ethyl ether to give a white powder and then lyophilized.

The crude peptide was purified by RP-HPLC on a semi-preparative C18-bonded silica column (Phenomenex, Kinetex 100Å, 100 × 21.2 mm, 5 µm), with detection at 214 and 220 nm. The flow rate was set to 17 mL min^-1^ with mobile phases A: 0.1% TFA in H_2_O v/v and B: ACN plus 0.1% TFA with a linear gradient starting from 5 to 40% B in 17 min. Analytical purity and retention time of synthesized peptide were determined using RP-HPLC-UV and the analogue showed >98% purity when monitored at 214 nm (Figure S1A). The exact mass of synthesized peptide was acquired on a SolariX XR FT-ICR 7T (Bruker Daltonics, Bremen, Germany). Sample was infused at 4 uL/min by a Hamilton Syringe. MS detection was operated in positive ionization mode with an ESI Apollo II source with the following parameters: drying temperature, 200°C; nebulizing gas flow (N_2_), 1 L/min; drying gas pressure, 4 L/min. Full scan MS data were acquired in the range of 100-1500 m/z, accumulation time, 0.030 ms at 1M. For MS/MS experiments, precursor ions were isolated with a 3Da width, with a collision energy of 15 eV; ion accumulation, 150 ms (Figure S1B).


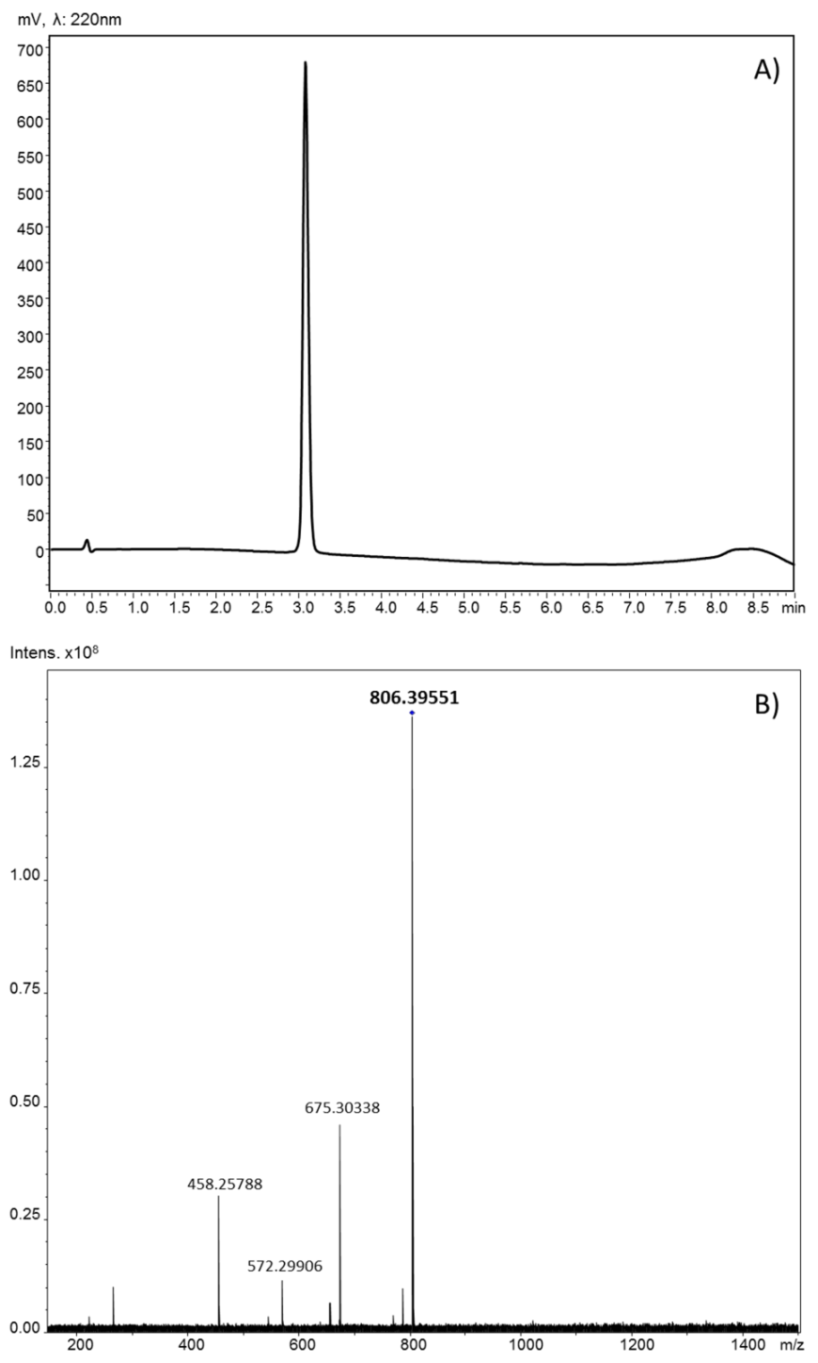


**Figure S1**: (A) Chromatographic profile acquired by RP-UHPLC-UV and (B) mass spectrum of synthetic BRP2 obtained by direct infusion Fourier Transform Ion Cyclotron Resonance MS.

**Appendix S2. Quantification of** **BRP2** **by UHPLC-MS/MS**

UHPLC-MS/MS analysis were performed on a Shimadzu Nexera UHPLC system (Shimadzu, Milan, Italy) consisting of two LC-30AD pumps, a SIL-30AC autosampler, a CTO-20AC column oven, a CBM-20A controller. The instrument was coupled online with a LCMS-8050 (Shimadzu, Kyoto, Japan) equipped with an electrospray source (ESI). LC-MS data elaboration was performed by the Labsolutions® software (Shimadzu).

The separation was carried on a on BIOshell™ A160 Peptide ES C18 100 × 2.1 mm × 2.7 μm (Supelco, Bellefonte PA, USA) employing as mobile phase A) H_2_O and B) ACN both acidified by formic acid 0.1% v/v, with the following gradient: 0-7.0 min, 5-40% B; 7-7.01 min, 40-95% B; 7.01-9.0 min, isocratic to 95% B. The flow rate was set to 0.5 mL min^-1^. Column oven was set to 35°C and 5 µL of samples were injected.

MS/MS analysis was conducted in selected reaction monitoring (SRM), employing the following transitions with 806.20 m/z as precursor: 806.20-458.45 m/z (quantifying ion); 806.20-349.25 m/z; 806.20-572.50 m/z. Dwell time: 50 ms, Interface temperature: 250°C, Desolvation line temperature: 200°C, Heat Block temperature: 300°C, Heating gas flow: 10 L/min, Nebulizing gas flow: 3.0 L/min.

For the quantification of BRP2 in buffalo ricotta digest and in BRF2, was employed as external standard the synthetic peptide. Stock solution was prepared in water, the calibration curve was obtained in a concentration range of 125-0.1 µg L^-1^ with eight concentration levels and triplicate injection of each level were run. Peak areas of BRP2 were plotted against corresponding concentrations. Linear regression was used to generate calibration curve (y = 0.0004x – 1.5321) with R^2^ values was ≥ 0.9998.

Instead, for the quantification of BRP2 in donor and receiver chamber, the stock solution was diluted with fresh HBSS to a series of concentrations ranged of 125-0.5 nM to establish the calibration curve (y = 0.000001x – 0,000363; R^2^ ≥ 0.9997).

**Appendix S3. Calculations of apparent permeability coefficient of BRP2**

The apparent permeability values of BRP2 were calculated according to the Eq. 1. This calculation requires that the sink conditions are fulfilled, that is, the ratio C_R_/C_D_ is less than 0.1 at each sampling point used in the analysis. The analysis is easily improved by considering the change of *C_D_.* The donor concentration was recalculated by subtracting the cumulative amount transported to the receiver chamber for each time interval:

*C_D_* (*t_i_*) = *C_D_* (*t_i–1_*) – $\frac{\left[ C_{R}\left( t_{i} \right) - f \times C_{R}\left( t_{i-1} \right) \right] \times V_{R}}{V_{D}}$

(2)

*C_D_* (*t_i_*) and *C_R_* (*t_i_*) (μM): donor and receiver chamber concentrations calculated at each sample occasion (*i*) from the donor and receiver concentrations at the previous occasion *C_D_* (*t_i–1_*) and *C_R_* (*t_i–1_*), respectively; *f=1–V_S_/V_R_*: sample replacement diluition factor; *V_S_* (cm^3^)*:* sample volume; *V_R_* (cm^3^)*:* receiver chamber volume; *V_D_* (cm^3^): donor chamber volume. ^[28]^

Integration of Eq.1 then gives:

*FA_cum_ = P_app_ × t_i_ =* $\frac{1}{A}$ *×* $\sum_{k=1}^{i} \frac{\left[ M_{Rk} \right]}{\left[ C_{D}\left( t_{k-1} \right)+ C_{D}\left( t_{k} \right) \right]/2}$*=* $\frac{1}{A}$ *×* $\sum_{k=1}^{i} \frac{\left[ C_{R}\left( t_{k} \right) - f \times C_{R}\left( t_{k-1} \right) \right] \times V_{R}}{\left[ C_{D}\left( t_{k-1} \right)+ C_{D}\left( t_{k} \right) \right]/2}$

(3)

*FA_cum_* (cm)*:* cumulative fraction transported; *t_i_:* the time point for the sampling occasion *i*; *t_k:_* the time point for the sampling occasion *k.*

The mass balance is calculated according to:

recovery (%) = (*C_D(fin)_ × V_D_ +*∑ (*C_S(t)_ × V_S(t)_*) + *C_R(fin)_ × V_R(fin)_*) *×* 100 / (*C_D(0)_  × V_D(0)_*)

(4)

Concentrations of sample on the donor (*C_D_*) and receiver (*C_R_*) sides of the monolayer at the start (0) or end (fin) of the experiment; *C_S(t)_* denotes the concentrations of the sample withdrawn at different points t. Our results showed that a mass balance values of BRP2 are more than 80%.





**Figure S2:** Transport of BRP2 across Caco-2 cell monolayer.

| **BRP2 (μM)** | ***P_app_* (10^-6^ cm/s)** |
| --- | --- |
| 1 | 0.53 ± 0.15 |
| 10 | 0.53 ± 0.01 |
| 50 | 0.20 ± 0.05 |
| 100 | 0.27 ± 0.07 |
| The time point was at 120 min. Data are means ± S.D. | |

**Table S1**: The apparent permeability coefficient (Papp) values of BRP2 with different concentrations.


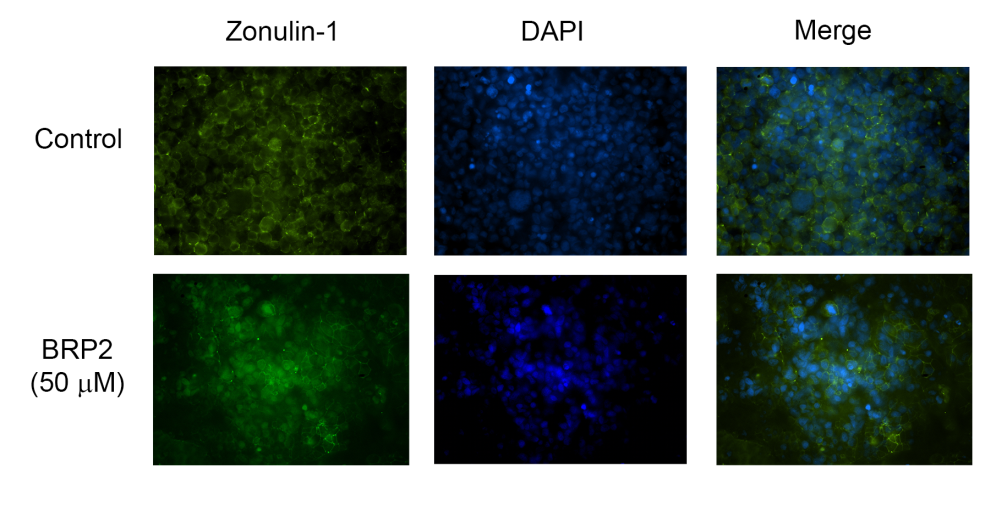


**Figure S3:** Fluorescence micrograph of the Caco-2 cell monolayers. Caco-2 cell monolayers treated with BRP2 (50 µM, 2 h) from TEER experiments were stained for tight junction protein expression of zonulin-1 (FITC, green). Nuclei were counterstained with DAPI (blue). Pictures are representative of two independent experiments. Original magnification 200X.

**Appendix S4. Database-driven bioactivity research**

The biological properties of BRP2 were not known as demonstrated by a database-driven bioactivity assessment such as: BIOPEP (http://www.uwm.edu.pl/biochemia/index.php/pl/biopep), EROP-Moscow (http://erop.inbi.ras.ru/), PeptideDB (http://www.peptides.be/), milkAMP (http://milkampdb.org/home.php), Antimicrobial Peptide Databases (APD2) (http://aps.unmc.edu/AP/main.php) and, Collection of AntiMicrobial Peptide (CAMP) (<http://www.camp.bicnirrh.res.in/>)

**Appendix S5. Effect of BRP2 on RAC1 and NADPH oxidase expression in intestinal epithelial cell line**


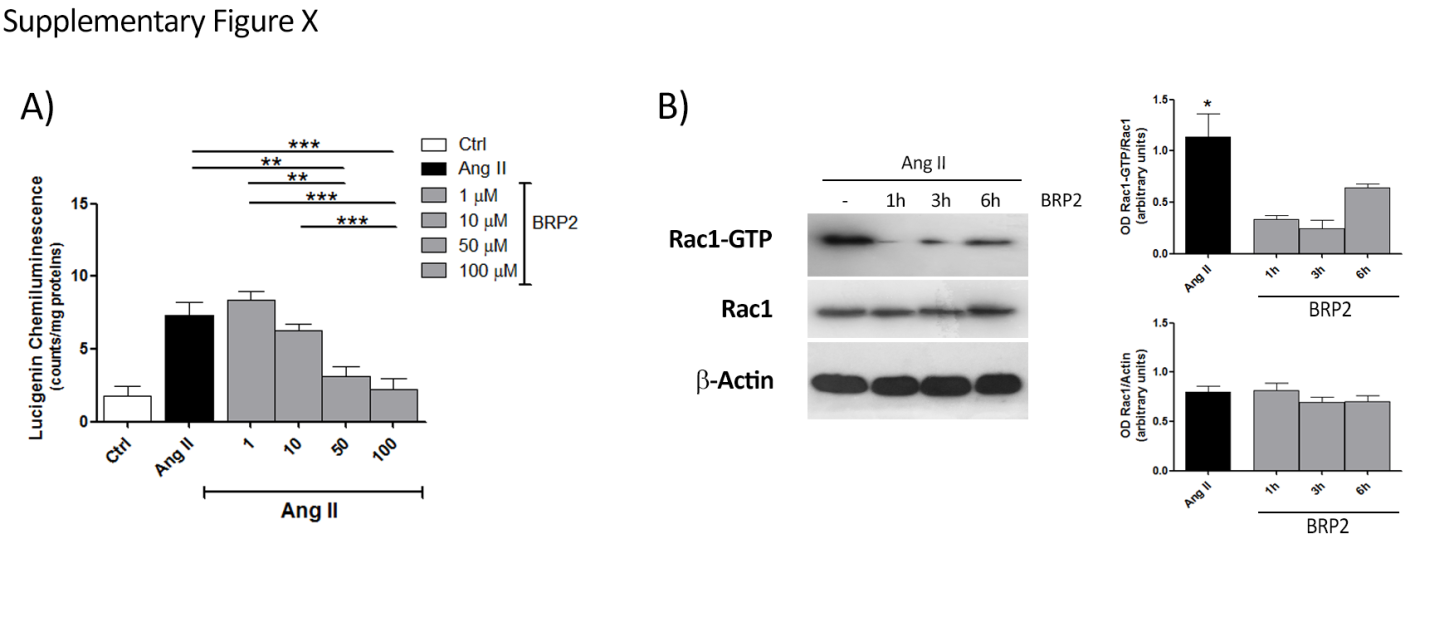


**Figure S4**: (A) Graphs of NADPH superoxide production in IEC-6 cells measured continuously in presence or absence of BRP2 by using 5 µmol L^-1^ lucigenin-enhanced chemiluminescence. Values are mean ± s.e.m., expressed as counts/(mg proteins). (n=4). (B) Representative immunoblot of three independent experiments from pull-down assay of IEC-6 cells for active Rac1 (Rac1-GTP), Ang II: 10^-5^ M, BRP2: 100 µM. (n = 4).
